# Efficacy and safety of baclofen in the prophylaxis of postoperative nausea and vomiting after laparoscopic sleeve gastrectomy: a randomized controlled trial

**DOI:** 10.1186/s12893-026-03696-4

**Published:** 2026-04-15

**Authors:** Aya Gamal Moussa, Sahar Mohammed El-Haggar, Tamer Mosaad El-Mahdy, Tarek Mohamed Mostafa

**Affiliations:** 1 Department of Clinical Pharmacy, Faculty of Pharmacy, Tanta University, Tanta, 31111 Egypt; 2 Department of General Surgery, Faculty of Medicine, Tanta University, Tanta, Egypt

**Keywords:** Laparoscopic sleeve gastrectomy, Postoperative nausea and vomiting, Baclofen, Substance P, Serotonin, Vasopressin

## Abstract

**Background:**

Postoperative nausea and vomiting (PONV) are among the most distressing aspects of the surgical experience, especially after laparoscopic sleeve gastrectomy (LSG). This study evaluated the efficacy and safety of baclofen for preventing PONV in patients with morbid obesity undergoing LSG.

**Methods:**

In this double-blind placebo-controlled parallel study, 100 morbidly obese patients scheduled for LSG were randomized into two groups: the control group (*n* = 50), which received placebo and the baclofen group (*n* = 50), which received 10 mg of oral baclofen one hour before the anticipated time of surgery. The severity of PONV was assessed using the Rhodes index, and the frequency of rescue analgesia and pain intensity during the first 48 h after anaesthesia were evaluated using a visual analogue scale (VAS). Serum levels of substance P, serotonin, and vasopressin at baseline and 24 h after surgery were also evaluated.

**Results:**

As compared to the control group, baclofen treated group exhibited significant decline in the severity of PONV during the first 24 h postoperatively (*P* = 0.001), a significantly lower frequency of rescue analgesia doses needed during the first 24 h postoperatively (*P* = 0.001), a significantly lower pain intensity at 0–2 h, 6 h and 12 h postoperatively (*P* = 0.001) and significantly lower serum levels of substance P, serotonin, and vasopressin (*P* = 0.001). Moreover, baclofen was safe and tolerable, and its implication was not associated with excessive sedation or extrapyramidal side effects.

**Conclusion:**

Baclofen could emerge as a promising new safe cost-effective agent for preventing PONV after LSG.

**Clinical trial registration:**

ClinicalTrials.gov Identifier: NCT05516953. Date of registration: 08/24/2022. URL: https://register.clinicaltrials.gov/prs/beta/studies/S000CF8D00000049/recordSummary.

## Background

According to the International Federation for the Surgery of Obesity and Metabolic Disorders (IFSO) 2022, data from 480,970 procedures performed across 25 countries showed that laparoscopic sleeve gastrectomy (LSG) was the most common bariatric procedure worldwide [1]. LSG is considered an effective single-stage procedure for morbidly obese patients with excellent and reliable outcomes and a negligible rate of complication [2]. 

Postoperative nausea and vomiting (PONV) remain the most common complication in the first 24 hours postoperatively despite the development of antiemetic medications and techniques [3, 4]. In the bariatric surgical population, the PONV rate is about 80% compared to 40% in the general surgical population [5]. The incidence of PONV after LSG was higher “66.9%” than after laparoscopic Roux-en-Y gastric bypass group"33.1%” [6]. Without preventive antiemetic treatment, the incidence of PONV after LSG can reach to about 80% [7]. Postoperative nausea and vomiting remain a major source of morbidity after laparoscopic bariatric surgery, contributing to complications such as prolonged hospitalization, delayed oral intake, readmissions, and higher healthcare costs. Despite various antiemetic strategies, PONV rates remain substantial and difficult to eliminate [8]. 

Optimal management of PONV is multifaceted, given the wide range of antiemetics with different pharmacokinetic properties, efficacy, and adverse-effect profiles. Selection of therapy should be individualized according to the clinical scenario, balancing prophylactic benefits against potential risks. At the institutional level, decisions are further shaped by cost-effectiveness, drug availability, and formulary policies. Although several guidelines currently exist, many of them focus only on specific patient groups, in addition to lack of comprehensive detail and being not fully updated in order to reflect the current evidence [7]. Moreover, few studies assessed the effect of antiemetic medications in the prophylaxis of PONV after LSG despite the popularity of LSG procedures and the lack of standardized protocol to effectively manage PONV [9]. 

Although 5-HT3 receptor antagonists are effective and safe for preventing PONV after laparoscopic surgery, their high cost is a significant limitation. Combination therapy using antiemetics with different mechanisms, often required for high-risk patients, further increases treatment expenses. Consequently, more cost-effective and efficient alternatives are still needed [10]. 

Baclofen is an agonist for gamma-aminobutyric acid (GABA) B receptors on pre- and postsynaptic neurons inside the central nervous system (CNS) and in peripheral nervous system [11]. Since the GABAB receptor-mediated signalling pathway is involved in emesis, baclofen may represent a novel therapeutic or prophylactic agent for PONV [12]. Importantly, several preclinical studies and limited clinical observations suggest antiemetic potential through GABA-B receptor mediated inhibition of emetic circuitry. Consistent with this, baclofen has been shown to reduce emesis and acid reflux episodes in neurologically impaired children with gastroesophageal reflux disease [12, 13]. Despite, nausea is listed among the most common adverse effects of baclofen, a clinical study in patients undergoing percutaneous nephrolithotomy reported no significant differences in postoperative vital signs or adverse effects between baclofen and placebo groups, supporting its safety [14]. These differences may reflect dose-dependent and population-specific effects, as well as the distinction between chronic therapy and single perioperative administration. In this context, this former knowledge encourages us to run this study to evaluate the efficacy of baclofen in the prevention of PONV in patients with morbid obesity who underwent LSG.

## Patients and methods

### Study design, and patients’ selection

In this double-blind, randomized, placebo-controlled, parallel study, one hundred patients with morbid obesity who underwent LSG were selected from the Gastrointestinal and Laparoscopic Surgery Unit, General Surgery Department, Tanta University Hospital, Tanta, Egypt, between August 2022 and December 2024. All participants gave their written informed consent. The investigator was provided with a sealed randomization code for each available medication generated by an independent researcher. Both the patients and the investigator were blind to the study medication. The participants were randomized using sealed envelope method into two groups; group 1 (the control group) which included 50 participants who were scheduled for LSG and received placebo (The rescue antiemetic, IV ondansetron 4 mg was administered for severe nausea, retching, or two or more emetic episodes that occurred during the first 48 h after surgery) and group 2 (the Baclofen group) which involved 50 participants who were scheduled for LSG and received 10 mg oral baclofen (Baclofen^®^; Al-Delta Pharmaceuticals Trading Company, Egypt) with a sip of water an hour before the anticipated time of surgery. Inclusion criteria were adults aged 18–60 years with morbid obesity undergoing LSG (BMI ≥ 35 kg/m² with comorbidities or ≥ 40 kg/m² without). Exclusion criteria included BMI > 55 kg/m², prior bariatric procedures, psychiatric illness, recent antiemetic or steroid use, recent vomiting, alcohol or drug abuse, drug hypersensitivity, and pregnancy or lactation.

A placebo-controlled design was adopted to evaluate the independent prophylactic effect of baclofen. All patients were closely monitored postoperatively, and predefined rescue antiemetic therapy was administered promptly in cases needed it, ensuring that no patient was denied appropriate treatment.

### Methods

#### Demography, history, physical and clinical examinations

All participants were submitted to demography, physical examination, family and medical history review, weight and height measurements and calculation of body mass index (BMI) according to the formula: (BMI) = [Weight (kg) ÷ Height (m^2^)]. Moreover, all participants were submitted to psychological and cardiological consultations and controlling comorbidities such as hypertension and diabetes.

#### Routine investigations needed for sleeve gastrectomy

Before surgery, all patients underwent routine laboratory and diagnostic evaluations, including CBC, inflammatory ratios (neutrophil/lymphocyte ratio (NLR) and platelet/lymphocyte ratio (PLR)), fasting glucose, lipid profile, coagulation tests (PT, PTT, INR), thyroid and liver function tests, kidney function tests, viral markers, pregnancy testing for females with reproductive age, pulmonary function testing, abdominal ultrasound, and chest X-ray.

#### Preoperative nausea and vomiting risk assessment

Preoperative nausea and vomiting risk assessment was calculated using the Apfel simplified score, which includes female gender, history of PONV and/or motion sickness, non-smoking status, and postoperative use of opioids [15]. 

#### General anaesthesia

All patients were informed about the anaesthesia process and were fasted for at least 8 h preoperatively. Anaesthesia, antibiotics, and postoperative analgesia were standardized to isolate the effects of the studied antiemetic. Baseline vital signs were recorded, and IV access was established. Patients received Ringer’s lactate (10–15 mL/kg TBW), followed by oxygenation via face mask for 3 min. Anaesthesia induction included: Fentanyl (1–2 µg/kg LBW), Propofol (1.5–2 mg/kg IBW), Rocuronium (1.2 mg/kg IBW). Maintenance was achieved with: Isoflurane (1.5–3%), Remifentanil infusion (0.1–0.4 µg/kg/min LBW). Ventilation was adjusted to maintain end-tidal CO₂ at 35–40 mmHg. Tramadol (100 mg IM) was given 15 min before surgery ended. Reversal of anaesthesia included neostigmine (70 µg/kg) and atropine (40 µg/kg). All patients received prophylactic Ceftriaxone (2 g IV).

#### Surgical technique

The technique used for LSG was described by Baltasar et al., 2005 [16]. Anaesthesia and surgery durations were documented. Pain was managed with IV paracetamol (500 mg) as needed. Patients were then observed in the surgical ward for 48 h.

#### PONV and pain assessments

PONV episodes (nausea, retching, and vomiting) were recorded during the first 48 h after anaesthesia in three-time intervals (0–2 h, 2–24 h, and 24–48 h). Severity was assessed using the Rhodes Index (RINVR), based on an eight-item questionnaire and each item scored 0–4 [17]. Rescue antiemetic protocol (Rescue ondansetron 4 mg IV) was given for cases with severe symptoms or for those with two or more emetic episodes, and the number of rescue doses was documented.

Pain intensity using visual analogue scores (VAS), adverse effects (dizziness, headache, drowsiness), and postoperative sedation scores (0–3) were also recorded at the same time points as PONV assessments.

#### Biochemical assays

Venous blood samples were collected 24 h after surgery, centrifuged, and the separated serum were stored at − 80 °C. Serum levels of substance P, serotonin, and vasopressin were later measured using ELISA kits (Sun red Biological Technology, China) according to the manufacturer’s instructions.

#### Primary and secondary outcomes

The primary outcome is the incidence of the complete response (absence of PONV and the lack of the need for rescue antiemetic therapy) within the first 24 h after anaesthesia. The secondary outcome is the change in the measured biological parameters (substance P, serotonin, and vasopressin).

#### Sample size calculation

G*Power software version 3.1 was used to calculate the required sample size. With a significance level (α-error) of 0.05, it is estimated that a total sample size of 100 patients (50 patients in each group) would have a statistical power of 80% to detect a medium effect size of 0.57 in the proportion of patients who experience PONV after 24 h using the Rhodes index between the two study groups based on a previous study [18].

#### Statistical analysis

The statistical analysis was conducted using SPSS statistical package version 27.0 (December 2020) IBM corporation software group, USA. The data were tested for normality using the Kolmogorov–Smirnov test or the Shapiro Wilk test. The mean values within the same group before and after surgery were compared using the paired student *t*-test. For normally distributed data, the mean values between the two groups before and after the 24 h of surgery were compared using an unpaired student *t*-test. Categorical variables were compared between groups using the Chi-Squared test. Risk ratios (RR) with 95% confidence intervals were calculated for categorical outcomes to provide an estimate of the magnitude and clinical relevance of the treatment effect. Fisher exact test was used for statistical analysis of any reported adverse effects. All data are expressed as mean ± SD, number and percentage (%). Correlations were assessed using Pearson’s coefficient depending on data distribution. All tests were two-tailed, and the significance level was set at *p* ≤ 0.05.

## Results

**Fig. 1:**
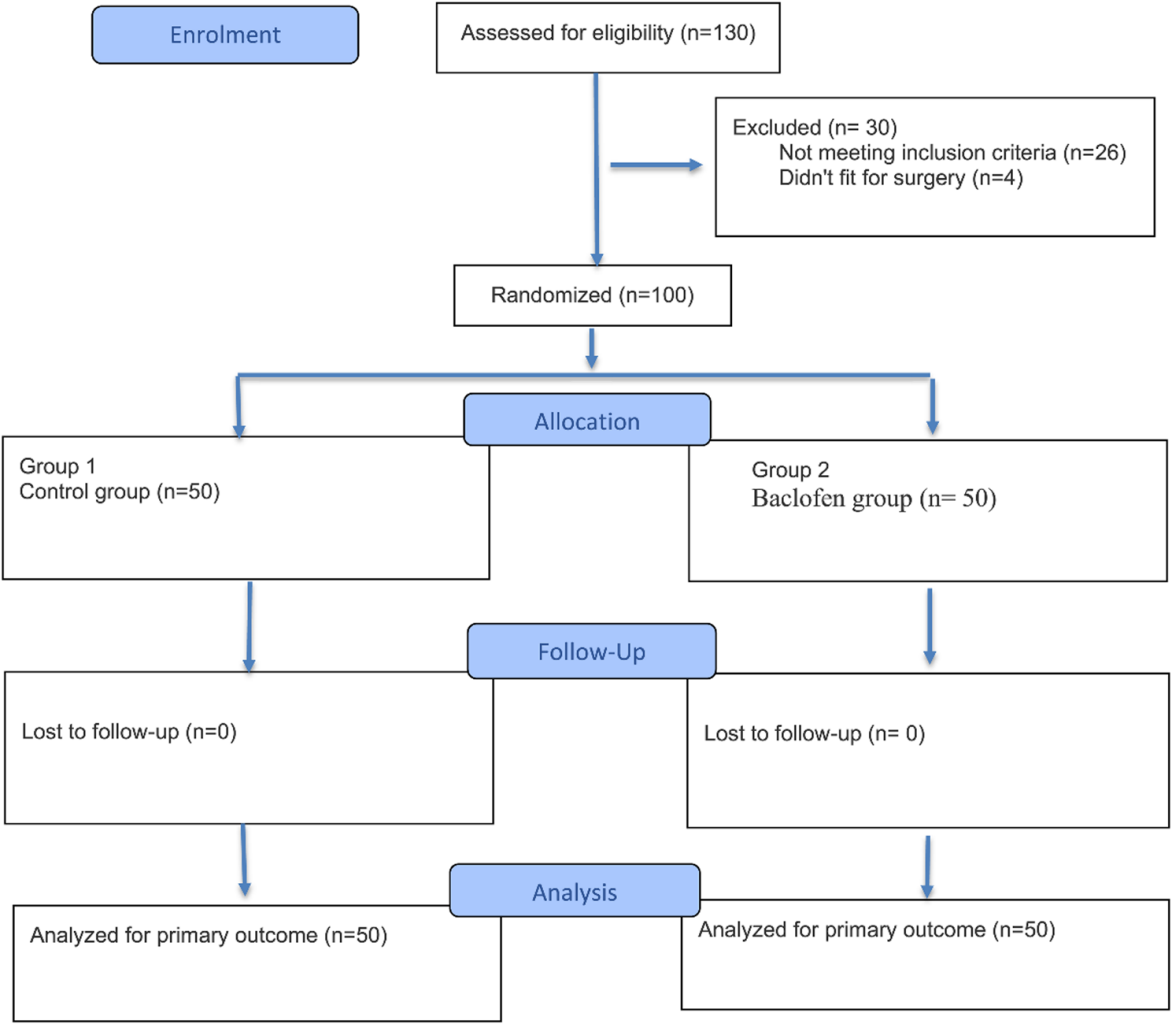
Flow diagram of study participants

Out of the 130 patients screened for eligibility, 26 patients didn’t meet the inclusion criteria, and 4 patients were not fit for surgery. In this context, the remaining 100 patients were randomized into the two study groups as shown in Fig. 1.

### Demographic characteristics, laboratory and clinical data

The baseline demographic, laboratory and clinical data for the two research groups were significantly similar (*P* > 0.05). There was no significant difference between the two study groups concerning age, gender, weight, height, BMI, laboratory data, previous surgical operation and comorbidities as postulated in Table 1.

**Table 1:** Baseline demographics and clinical data

Variables	Control group (*n* = 50)	Baclofen group (*n* = 50)	*P*-value
Age (years)	28.50±5.21	29.62±5.59	0.303
Gender			
Male Female	4 (8%) 46 (92%)	4 (8%) 46 (92%)	1.0
Weight (Kg)	102.45±7.34	101.32±9.10	0.496
Height (cm)	160±4	158±5	0.215
BMI (Kg/m^2^)	40.50±2.75	38.90±2.69	0.165
Hemoglobin (gm/dl)	13.2 ± 2.16	13.1 ± 3.12	0.853
Leukocytes count (x10^9^/L)	8.1 ± 3.12	8.2 ± 2.69	0.864
Neutrophil count (x10^9^/L)	4.41 ± 3.17	4.53 ± 3.19	0.851
Lymphocyte count (x10^9^/L)	2.76 ± 1.72	2.78 ± 1.69	0.953
Platelet count (x10^9^/L)	280 ± 98.53	292 ± 102.30	0.552
Neutrophil/lymphocyte ratio	1.07 ± 0.42	1.08 ± 0.47	0.911
Platelet/lymphocyte ratio	127.9 ± 58.45	128.2 ± 61.23	0.980
Previous surgical operations			
- Appendectomy - Cholecystectomy - Cesarean section - Tonsillectomy - Ovarian cystectomy	2 3 5 5 4	2 4 4 5 4	1.0 0.695 0.727 1.0 1.0
Total	19 (38%)	19 (38%)	1.0
*Comorbidities:*			
- Hypertension - Diabetes - Arthritis - Sleep apnea	2 5 10 1	3 4 8 1	0.646 0.727 0.603 1.0
Total	18 (36%)	16 (32%)	0.673

Data are presented as mean ± SD, number and percentage (%)

*Kg* Kilogram, *cm* Centimetre, *BMI* Body mass index, *m*^2^ meter square, *gm/dl* Gram per decilitre, *L* liter

The significance level was set at *P*≤ 0.05

### Risk assessment for PONV and surgery-related parameters

There were no significant differences (*P* > 0.05) between both study groups regarding the four risk factors for the Apfel simplified score which includes female gender, non-smoking status, history of PONV and/or motion sickness, postoperative use of opioids, and risk score for PONV as well as surgery related parameters as shown in Table 2.

**Table 2:** Risk assessment for PONV and surgery-related parameters

Risk factors	Control group (*n* = 50)	Baclofen group (*n* = 50)	*P*-value
1-Gender			
Male Female	4 (8%) 46 (92%)	4 (8%) 46 (92%)	1.0
2-Smoking status			
Smoker Nonsmoker	2 (4%) 48 (96%)	3 (6%) 47 (94%)	0.646
3-History of PONV and/or motion sickness			
Yes No	17 (34%) 33 (66%)	15 (30%) 35 (70%)	0.668
4-Use of postoperative opioids			
Yes No	0 (0%) 50 (100%)	0 (0%) 50 (100%)	-
Risk score for PONV			
0 1 2 3 4	0 0 34 (68%) 16 (32%) 0	0 1 (2%) 34 (68%) 15 (30%) 0	0.597
Surgery related parameters			
Duration of anaesthesia (min)	82 ± 12.32	80 ± 13.84	0.447
Duration of operation (min)	52 ± 18.32	55 ± 16.94	0.397
Duration of insufflation (min)	40 ± 15.19	43 ± 14.94	0.382

Data are presented as mean ± SD, number and percentage (%)

*min* Minute

Significance level was set at *P*≤ 0.05

### Postoperative pain assessment

The comparison of pain intensity for the two study groups at 0–2 h, 6 h, and 12 h revealed that, the intensity of pain was significantly lower in the baclofen group than the control group (*P* = 0.001). However, at 24 h, and 48 h, there were no significant differences regarding the intensity of pain between the two study groups (*P* > 0.05) as shown in Table 3.

**Table 3:** Postoperative pain assessment for the two study groups

Pain score	Control group (*n* = 50)	Baclofen group (*n* = 50)	*P*-value	RR (95%CI)
	No. (%)	No. (%)		
a-0–2 h				
-No pain -Mild. - Moderate -Severe	9 (18%) 30 (60%) 11 (22%) 0	41(82%) 7 (14%) 2 (4%) 0	0.001*	0.148 (0.031–0.706)
b- 6 h				
-No pain -Mild -Moderate -Severe	0 14 (28%) 29 (58%) 7 (14%)	14 (28%) 29 (58%) 6 (12%) 1 (2%)	0.001*	0.326 (0.185–0.742)
c- 12 h				
-No pain -Mild -Moderate -Severe	0 15 (30%) 30 (60%) 5 (10%)	5 (10%) 35 (70%) 8 (16%) 2 (4%)	0.001*	0.107 (0.043–0.269)
d- 24 h				
-No pain -Mild -Moderate -Severe	20 (40%) 25 (50%) 5 (10%) 0	26 (52%) 23 (46%) 1 (2%) 0	0.171	0.184 (0.021–1.633)
e- 48 h				
- No pain - Mild - Moderate -Severe	30 (60%) 18 (36%) 2 (4%) 0	34 (68%) 14 (28%) 2 (4%) 0	0.687	0.254 (0.102–7.392)
Time for the first analgesic request (h)				
0–2 h < 2–6 h < 6–12 h < 12–24 h	18 (36%) 32 (64%) 0 0	0 9 (18%) 39 (78%) 2 (4%)	0.001*	2.415 (1.684–3.914)
Type of postoperative analgesic used and dose.				
1-Acetaminophen alone (500 mg/6 h)	28 (56%)	44 (88%)	0.001*	0.415 (0.218–0.742)
2- Acetaminophen plus ketolac (30 mg/2 ml). - One dose. - Two doses.	19 (38%) 8 (16%) 11 (22%)	3 (6%) 2 (4%) 1 (2%)	0.001* 0.046* 0.001*	1.745 (1.085–2.103) 1.320 (1.141–1.532) 1.632 (1.164–1.873)
3- Acetaminophen plus morphine sulfate 10 mg/ml solution (0.1 mg/kg).	3 (6%)	0	0.078	1.321 (0.748–1.843)

Data are presented as numbers and percentages (%)

The Visual Analogue Scale measures pain intensity as follow; no pain (0); mild (1–3), moderate (3–5), severe (5–7), very severe (7–9)

*h* Hour, *mg* Milligram, *mg/kg* Milligram per kilogram

The significance level was set at *P*≤ 0.05

* Statistically significant difference

Also, the time for the first analgesic request was significantly longer in the baclofen group as compared to the control group (*P* = 0.001), and the frequency of rescue analgesia doses needed was significantly lower in in the baclofen group as compared to the control group (*P* = 0.001) as shown in Table 3.

Furthermore, the use of acetaminophen alone and the use of combined analgesics consisting of acetaminophen plus ketolac were significantly lower in the baclofen group as compared to the control group (*P* = 0.001) as postulated in Table 3.

### Postoperative sedation assessment

The comparison of sedation levels using the Ramsay Sedation Score for the two research groups showed that, the sedation scores at 0–2 h and 2–24 h were significantly higher in the baclofen group as compared to the control group (*P* = 0.001). However, at 24–48 h, the sedation scores were statistically similar for the two study groups since all patients were fully conscious as shown in Table 4.

**Table 4:** Postoperative sedation score assessment

Sedation score	Control group (*n* = 50)	Baclofen group (*n* = 50)	*P*-value	RR (95%CI)
	No. (%)	No. (%)		
0–2 h:				
Score 1 Score 2 Score 3 Score 4	38 (76%) 10 (20%) 2 (4%) 0 (0%)	0 (0%) 35 (70%) 11 (22%) 4 (8%)	0.001*	2.054 (1.416–2.631)
< 2–24 h:				
Score 1 Score 2	34(68%) 16(32%)	8(16%) 42(84%)	0.001*	1.674 (1.352–1.832)
< 24–48 h				
Score 1	50 (100%)	50 (100%)	-	

Data are presented as number and percentage (%)

h: hour; Score 1: The patient is anxious; Score 2: The patient is cooperative, oriented, and tranquil; Score 3: The patient responds to commands only; Score 4: The patient exhibits a brisk response to a light glabellar tap or loud auditory stimulus

Significance level was set at P≤ 0.05

*Statistically significant difference

### Postoperative retching, nausea, and vomiting

The assessment of the severity of post-operative nausea and vomiting using the Rhodes index showed that, in the first 24 h after surgery, patients in baclofen group exhibited significantly lower retching, nausea and vomiting scores than patients in group 1 (*P* = 0.001). However, there was no significant difference between patients in both study groups regarding the development of retching, nausea and vomiting episodes within 24–48 h after surgery. Additionally, the total rescue antiemetics in the first 24 h were significantly higher in the control group than the baclofen group (*P* = 0.001) as postulated in Table 5.

**Table 5:** Severity of postoperative nausea and vomiting during the first 48 hours after surgery

Time in hours & Scores	Control group (*n* = 50)	Baclofen group (*n* = 50)	*P*-value	RR (95%CI)
-0–2 h				
No Mild Moderate Great Severe	21 (42%) 16 (32%) 10 (20%) 3 (6%) 0	50 (100%) 0 (0%) 0 (0%) 0 (0%) 0	0.001*	0.452 (0.218–0.913)
Rescue antiemetics	10 (20%)	0 (0%)	0.001*	1.304 (1.017–1.862)
-2–24 h				
No Mild Moderate Great Severe	18 (36%) 15 (30%) 12 (24%) 4 (8%) 1 (2%)	49 (98%) 0 (0%) 1 (2%) 0 (0%) 0 (0%)	0.001*	0.632 (0.417–0.843)
Rescue antiemetics	16 (32%)	1 (2%)	0.001*	1.325 (1.174–2.065)
Total Rescue antiemetics in the first 24 h	26 (52%)	1 (2%)	0.001*	1.418 (1.206–1.854)
-24–48 h				
No	50 (100%)	50 (100%)	-	

Data are presented as number and percentage (%)

The severity of PONV was assessed based on the experience scores of RINVR (Rhodes index of nausea, vomiting and retching) as follows: No: 0; Mild:1–8; Moderate: 9–16; Great: 17– 24; Severe :25–32

Significance level was set at *P*≤ 0.05 ; * Statistically significant difference

### Effect of intervention on the assessed biological markers

At baseline (Preoperative), the serum levels of substance P, serotonin and vasopressin didn’t differ significantly between the two study groups (P_2_ > 0.05), as shown in Table 6.

**Table 6:** Change in the levels of biological markers for the two study groups

Biological markers	Control group (*n* = 50)	Baclofen group (*n* = 50)	*P*_2_-Value
Substance P (ng/L)			
Pre-operative Post-operative	221.48 ± 99.12 333.77 ± 187.69	225.46 ± 80.10 129.82 ± 39.69	0.818 0.001^*^
P_1_-Value	0.001^*^	0.001^*^	
Serotonin (ng/ml)			
Pre-operative Post-operative	21.88 ± 17.11 87.50 ± 28.46	19.56 ± 10.36 15.12 ± 7.86	0.347 0.001^*^
P_1_-Value	0.001^*^	0.018^*^	
Vasopressin (Pg/ml)			
Pre-operative Post-operative	14.13 ± 4.79 57.70 ± 11.80	14.74 ± 4.08 15.60 ± 3.62	0.513 0.001^*^
P_1_-Value	0.001^*^	0.265	

Data are presented as mean ± SD

*ng/L* Nanogram per liter, *ng/ml* Nanogram per millilitre, *pg/ml* Picogram per millilitre

The significance level was set at *P*≤ 0.05

P1-value: difference within the same group (before versus after surgery)

P2-value: difference between the two groups

*Statistically significant difference at *P*< 0.05

After surgery and as compared to baseline data, the control group showed significant elevation of the serum levels of substance P (P_1_ = 0.001), serotonin (P_1_ = 0.001) and vasopressin (P_1_ = 0.001). On the other hand, after surgery and as compared to the baseline values, the baclofen group showed significant reductions in the serum levels of substance P (P_1_ = 0.001) and serotonin (P_1_ = 0.018) which was associated with non-significant variation in the serum level of vasopressin (P_1_ = 0.265) as postulated in Table 6.

The comparison between the two study groups after surgery revealed that the baclofen group showed a significant decline in the serum levels of substance P (P_2_ = 0.001), serotonin (P_2_ = 0.001) and vasopressin (P_2_ = 0.001) when compared to the control group as postulated in Table 6.

### Correlation analysis

The correlation analysis conducted after surgery between the severity of PONV at the period from 2 to 24 h and measured biological markers for both groups revealed the existence of significant positive correlations between the severity of PONV and the serum levels of substance P (*r* = 0.921; *P* = 0.001 and *r* = 0.446; *P* = 0.001 for the control and the baclofen group respectively), serotonin, (*r* = 0.976; *P* = 0.001 and *r* = 0.624; *P* = 0.001 for the control and the baclofen group respectively) and vasopressin (*r* = 0.969; *P* = 0.001 and *r* = 0.485; *P* = 0.001 for the control and the baclofen group respectively).

### Safety and tolerability of the study medications

The adverse effects observed in both study groups were mild and controllable. There were no significant differences between the control group and the baclofen group regarding the reported adverse effects including headache [7 patients (14%) versus 8 patients (16%); *P* = 0.779], dizziness [2 patients (4%) versus 1 patient (2%); *P* = 0.558], diarrhea [0 patient (0%) versus1 patient (2%); *P* = 0.315], dry mouth [12 patients (24%) versus 11 patients (22%); *P* = 0.812] and joint pain [9 patients (18%) versus 12 patients (24%); *P* = 0.461]. No one in both groups developed excessive sedation or extrapyramidal symptoms.

## Discussion

PONV remain significant clinical challenges following general anaesthesia, with the highest incidence typically occurring within the first 6 h after surgery in the absence of prophylactic therapy [19]. PONV is particularly prevalent after bariatric procedures and LSG is considered among the most emetogenic standard bariatric operations [6, 20]. Despite multiple preventive strategies, optimal management remains complex secondary to the multifactorial pathophysiology of PONV [21]. 

Antiemetic selection is guided by its efficacy, safety, cost, and dosing convenience. Safety concerns include QTc prolongation with butyrophenones and first-generation 5-HT3 receptor antagonists. Furthermore, pharmacogenetic variability may influence drug response [19]. Baclofen is an agonist for GABA B receptors which is one of the numerous neuronal proteins that have emerged as potential targets of inhaled anaesthetics that are considered one of the primary causes for PONV [11, 22]. Baclofen has a good oral bioavailability (~ 70–85%) and the time needed to reach the peak concentration after oral administration (T max) was reported to be 1 h, 1.13 h and 1.2 h according to the findings of other authors [23–26]. In this context, during the current study, baclofen was administered orally 60 min before induction of anaesthesia which seems sufficient to achieve the therapeutic plasma level during the intraoperative and early postoperative phases based on its rapid absorption and favourable bioavailability [23–26]. Furthermore, laparoscopic sleeve gastrectomy does not impair preoperative absorption, as drug intake occurred 1 h before gastric resection.

Although baclofen has a relatively short elimination half-life (2–6 h), its clinical effects are not solely determined by plasma concentration. Central modulation of brainstem emetic pathways may extend beyond its pharmacokinetic elimination when administered preoperatively [27, 28]. Because PONV is multifactorial and commonly assessed over 24 h in randomized trials and consensus guidelines extended monitoring were undertaken to determine whether early neuromodulation translated into sustained clinical benefit [7]. 

In the present study, baclofen was associated with a reduction in the severity of PONV and with a decrease in rescue antiemetic requirements. Mechanistically, GABA-B receptor activation within the nucleus of the solitary tract may attenuate efferent signalling from the vomiting center [12]. However, preclinical studies provide mechanistic insight; they cannot be directly extrapolated to complex clinical settings such as bariatric surgery. Our preliminary findings therefore represent clinical observations that complement, rather than directly derive from experimental data [12]. Evidence from neurologically impaired paediatric patients with gastroesophageal reflux disease also suggests that baclofen may reduce emesis frequency, although these data originate from a different clinical context [13].

Blood samples were withdrawn 24 h after surgery in order to evaluate and interpret short-half-life mediators (Substance P, Vasopressin and Serotonin). This was supported by other former studies suggested that, in patients who develop PONV, substance P levels remain unchanged at the end of anaesthesia, followed by a significant increase within 24 h. Conversely, in patients without PONV, substance P levels tend to decrease at the end of anesthesia and recover by 24 h [29]. Directly after surgery, postoperative stress and postoperative pain increase the secretion and the circulating level of vasopressin which generally tended to return to its normal level within approximately five days after surgery [30]. Also, other authors reported that, the serum level of serotonin was significantly higher in patients with nausea and vomiting than its serum level in patients without nausea and vomiting [31]. Given the short half-lives of these mediators, the 24-hour measurement likely reflects cumulative neurohumoral activation rather than transient fluctuations. Nonetheless, single time-point assessment limits mechanistic interpretation, and serial measurements would provide more robust insight in future studies.

Regarding the assessed serum biomarkers, a positive correlation was observed between PONV severity and substance P levels. Substance P is a recognized neuropeptide involved in emesis and nociception through NK1 receptor activation [32]. The reduction in serum substance P observed in the baclofen group may reflect both attenuation of emetic pathways and reduced postoperative pain. Importantly, substance P is a well-established mediator of pain; therefore, postoperative elevation may physiologically reflect nociceptive activation rather than a specific antiemetic mechanism. Previous reports have demonstrated increased plasma substance P in patients with PONV [29, 33].

Similarly, serotonin plays a central role in emesis via vagal afferent stimulation following release from enteroendocrine cells [22]. Reduced postoperative serotonin levels in the baclofen group and the observed correlation with the severity of PONV come in alignment with some previous findings [31]. Although contradictory results have also been reported [34]. Variability in methodology, timing of measurement, and population characteristics may explain these inconsistencies. Despite serotonin modulation may contribute to the observed clinical effect, it should not be interpreted as a conclusive mechanistic proof.

Vasopressin levels were also positively correlated with the severity of PONV and were lower in the baclofen group. Surgical stress, pain, and abdominal insufflation with carbon dioxide during laparoscopic procedures are known to increase vasopressin secretion [30, 35, 36]. The observed reduction in vasopressin level may therefore reflect attenuation of stress and pain responses rather than a direct antiemetic pathway.

The observed reductions in the circulating levels of substance P, serotonin, and vasopressin in the baclofen group could be explained on the basis of the potential interaction between baclofen-mediated GABA-B receptor activation and neurochemical pathways involved in emesis regulation. Since these measurements represent observational associations within the clinical setting, causality cannot be established. Therefore, further mechanistic and experimental studies are required in order to clarify whether baclofen directly modulates these neurochemical mediators. Given the short half-lives of these mediators, the 24-hour measurement likely reflects cumulative neurohumoral activation rather than transient fluctuations. Nonetheless, single time-point assessment limits mechanistic interpretation, and serial measurements would provide more robust insight in future studies.

Regarding the strong positive correlations between the severity of PONV and the postoperative serum levels of the assessed biomarkers, the high values of correlation coefficient could reflect the well-established mechanistic role of these neurotransmitters in the emetic pathway, particularly through activation of vagal afferents nerves and emetic center in CNS. Furthermore, the study population included in the current study were a relatively homogeneous cohort of patients undergoing the same surgical procedure (LSG) with standardized anaesthesia and analgesic protocols, which may have reduced variability and strengthened observable associations. However, these correlations can’t establish causality and the high correlation coefficients may be overestimated secondary to the relatively small sample size.

Baclofen’s analgesic effect is mediated through presynaptic GABA-B receptor activation and reduced excitatory neurotransmitter release [37]. This may have contributed to reduced early postoperative pain and delayed need for rescue analgesia. Our former findings seem parallel to previously reported data revealed that baclofen may represent a useful adjuvant analgesic for the treatment of cancer pain [38]. Additionally, baclofen was recommended to be used for patients with non-adequate optimal multimodal analgesic techniques [39]. However, the analgesic benefit was limited to the early postoperative period in consistent with its pharmacokinetic profile. However, contradictory data were reported by other former studies [14]. These conflicting findings could be attributed to the influence of dose, population, and methodology which in turn underscore the need for cautious interpretation.

Although the spinal cord is the principal site of action for baclofen, its receptors are also found in the brain so binding to the presynaptic GABAB receptors within the brain stem can explain its CNS related side effects such as sedation. As compared to the control group, the sedation scores were higher in the baclofen group during the early postoperative period in consistent with its central mechanism of action [40, 41]. Importantly, this did not result in respiratory compromise, delayed ambulation, or prolonged hospitalization in our cohort. Nevertheless, caution is advisable in patients with severe obstructive sleep apnea. No significant differences in overall adverse effects were observed between groups, supporting the tolerability of a single low preoperative dose of baclofen [42, 43]. However larger study are still recommended to confirm safety of such drug. From a clinical perspective, incorporation of baclofen into Enhanced Recovery After Surgery (ERAS) pathways may be considered if its opioid-sparing and potential antiemetic effects are confirmed without clinically meaningful sedation [44]. Baclofen at a single oral dose of 10 mg preoperatively was reported to be safe and it has been reported to be safe when administered in obese patients with gastroesophageal reflux disease [14, 45, 46]. In our study, a single 10 mg oral dose was not associated with respiratory depression, hemodynamic instability, or delayed recovery, likely due to the low dose used. However, routine adoption should await validation in larger multicenter trials.

It is worth mentioning that, 42% of patients in the control group did not experience nausea or vomiting in in the early (0–2 h) postoperative period. This observation could be explained on the basis that, a substantial proportion of patients (76%) in the control group were sedated during the early postoperative period (0–2 h) which may have influenced subjective reporting of nausea and vomiting since patients are generally expected to regain responsiveness to stimulation within 60–90 min post-anaesthesia [47]. Another former study reported that, 9.46% of 443 surgical patients remained unresponsive for 15–90 min following general anaesthesia [48]. Therefore, assessment of PONV beyond the immediate recovery phase (6–24 h) may provide a more clinically representative evaluation.

The strengths of this study include its randomized double-blind placebo-controlled design and its evaluation of both clinical outcomes and related serum biomarkers in morbidly obese LSG patients. Limitations of the current study include its single-center design, restriction to LSG procedures, and the reliance on single time-point biomarker assessment. Also, no formal correction for multiple comparisons was conducted since we focused on one single primary outcome and our secondary outcomes were positively correlated with the occurrence of PONV, therefore multiple comparisons corrections were not applied in order to avoid decreasing the power and increasing type II error. Future multicenter studies with serial biomarker measurements and larger populations are warranted to confirm these preliminary findings, validate these biomarker associations and better define baclofen’s role within multimodal PONV prophylaxis strategies.

## Conclusion

Baclofen significantly reduced the severity of PONV, delayed the first analgesic request, lowered the need for rescue analgesia, and decreased serum levels of PONV related biomarkers (substance P, serotonin, and vasopressin). Although, baclofen was not associated with serious adverse effects and its reported adverse effects were not significantly different from those reported with the control group, the study was not designed to evaluate the safety profile of baclofen. In this context, larger multicentre trials are required in order to confirm the efficacy of baclofen against PONV and to detect the less frequent but clinically relevant adverse events and to confirm its safety profile in bariatric populations.

## Data Availability

The datasets used and/or analysed during the current study are available from the corresponding author on reasonable request.

## Abbreviations

PONV: Postoperative Nausea and Vomiting
LSG: Laparoscopic Sleeve Gastrectomy
BMI: Body Mass Index
VAS: Visual Analogue Scale
Rhodes Index: RINVR – Rhodes Index of Nausea, Vomiting, and Retching
GABA: Gamma-Aminobutyric Acid
GABA-B Receptor: A metabotropic receptor subtype for GABA
ELISA: Enzyme-Linked Immunosorbent Assay
PACU: Post-Anaesthesia Care Unit
CBC: Complete Blood Count
PT: Prothrombin Time
PTT: Partial Thromboplastin Time
INR: International Normalized Ratio
ALT: Alanine Aminotransferase
AST: Aspartate Aminotransferase
ALP: Alkaline Phosphatase
GGT: Gamma-Glutamyl Transferase
BUN: Blood Urea Nitrogen
S. Cr: Serum Creatinine
T3: Triiodothyronine
T4: Thyroxine
TSH: Thyroid-Stimulating Hormone

## References

[1] Kamal FA, Fernet L, Rodriguez M. Nutritional Deficiencies Before and After Bariatric Surgery in Low-and High-Income Countries: Prevention and Treatment. Cureus. 2024;16(2):1–13. 10.7759/cureus.55062 PMC10977614 38550458

[2] Gadelkarim A, Abdel Raheem O, Saleem AA. Outcomes of Sleeve Gastrectomy in Obese Patients: A Retrospective Study. Egypt J Hosp Med. 2023;90(2):3053–61.

[3] Zheng XZ, Cheng B, Luo J, Xiong QJ, Min S, Wei K. The characteristics and risk factors of the postoperative nausea and vomiting in female patients undergoing laparoscopic sleeve gastrectomy and laparoscopic gynecological surgeries: a propensity score matching analysis. Eur Rev Med Pharmacol Sci. 2021;25(1):182–9. 33506906 10.26355/eurrev_202101_24383

[4] Zengin M, Sazak H, Baldemir R, Aydemir S, Acar LN, Alagoz A. Parameters affecting nausea and vomiting after thoracoscopic wedge resection in patients with pneumothorax. Cureus. 2021;13(11):1–11. 10.7759/cureus.19926 PMC8710436 34966615

[5] Kwak H, Chang YJ, Lee KC, Jung WS, Kwon S, Jo YY. Antiemetic efficacy of dexmedetomidine versus dexmedetomidine-dexamethasone combination in patients undergoing breast surgery. J Int Med Res. 2019;47(10):5060–9. 31510871 10.1177/0300060519872031 PMC6833383

[6] Suh S, Helm M, Kindel TL, Goldblatt MI, Gould JC, Higgins RM. The impact of nausea on postoperative outcomes in bariatric surgery patients. Surg Endosc. 2020;34(7):3085–91. 31388805 10.1007/s00464-019-07058-5

[7] Gan TJ, Belani KG, Bergese S, Chung F, Diemunsch P, Habib AS, et al. Fourth consensus guidelines for the management of postoperative nausea and vomiting. Anesth Analg. 2020;131(2):411–48. 32467512 10.1213/ANE.0000000000004833

[8] Yimer H, Ayalew N, Abdisa Z, Aregawi A. Effect of sub-hypnotic dose of propofol on prevention of postoperative nausea and vomiting as part of multimodal antiemetic in patients undergoing open abdominal surgery: a prospective cohort study, Gondar University Hospital, Northwest Ethiopia, 2016.Int. J Surg Open. 2018;10:15–20.

[9] Halliday TA, Sundqvist J, Hultin M, Walldén J. Post-operative nausea and vomiting in bariatric surgery patients: an observational study. Acta Anaesthesiol Scand. 2017;61(5):471–9. 28374473 10.1111/aas.12884

[10] Kranke P, Diemunsch P. The 2014 consensus guidelines for the management of postoperative nausea and vomiting: a leapfrog towards a postoperative nausea and vomiting-free hospital. Eur J Anaesthesiol. 2014;31(12):651–3. 25350527 10.1097/EJA.0000000000000080

[11] Romito JW, Turner ER, Rosener JA, Coldiron L, Udipi A, Nohrn L, et al. Baclofen therapeutics, toxicity, and withdrawal: a narrative review. SAGE Open Med. 2021;9:1–13. 10.1177/20503121211022197 PMC8182184 34158937

[12] Konno K, Sugino S, Shibata T, Misawa K, Imamura-Kawasawa Y, Suzuki J, et al. Antiemetic effects of baclofen in a shrew model of postoperative nausea and vomiting: Whole-transcriptome analysis in the nucleus of the solitary tract. CNS Neurosci Ther. 2022;28(6):922–31. 35238164 10.1111/cns.13823 PMC9062569

[13] Kawai M, Kawahara H, Hirayama S, Yoshimura N, Ida S. Effect of baclofen on emesis and 24-hour esophageal pH in neurologically impaired children with gastroesophageal reflux disease. J Pediatr Gastroenterol Nutr. 2004;38(3):317–23. 15076634 10.1097/00005176-200403000-00017

[14] Mandabach M, Deichmann P, Massoll A, Graves S, Assimos D, Wood K, et al. Use of Baclofen Premedication as an Analgesic Adjuvant in Patients Undergoing Percutaneous Nephrolithotripsy: A Placebo-Controlled, Double Blind Randomized Trial. Cureus. 2024;16(7):1–7. 10.7759/cureus.64235 PMC11312425 39130904

[15] Apfel CC, Laara E, Koivuranta M, Greim CA, Roewer N. A simplified risk score for predicting postoperative nausea and vomiting: conclusions from cross-validations between two centers. Anesthesiolog. 1999;91(3):693–700. 10.1097/00000542-199909000-00022 10485781

[16] Baltasar A, Serra C, Perez N, Bou R, Bengochea M, Ferri L. Laparoscopic sleeve gastrectomy: a multi-purpose bariatric operation. Obes Surg. 2005;15(8):1124–8. 16197783 10.1381/0960892055002248

[17] Kushner BS, Freeman D, Sparkman J, Salles A, Eagon JC, Eckhouse SR. Assessment of postoperative nausea and vomiting after bariatric surgery using a validated questionnaire. SurgObesRelatDis. 2020;16(10):1505–13. 10.1016/j.soard.2020.05.017 32665115

[18] Ortiz E, González AI, Jaime V, Guzmán JA, Esparza I, Orozco JO, et al. The impact of Aprepitant on Nausea and Vomiting following Laparoscopic Sleeve Gastrectomy: A Blinded Randomized Controlled Trial. Obes Surg. 2024;34(4):1316–23. 38429485 10.1007/s11695-024-07129-0

[19] El Rabeey M, Shaboob I, Abo Gamaz A. Updates In Postopertive Nausea And Vomiting. Benha med j. 2022;39(1):46–61.

[20] Parisi A, Desiderio J, Cirocchi R, Trastulli S. Enhanced recovery after surgery (Eras): A systematic review of randomized controlled trials (RCTs) in bariatric surgery. Obes Surg. 2020;30(12):5071–85. 32981000 10.1007/s11695-020-05000-6

[21] Vilchis-Valentin D, García-Maldonado M, Larrazolo-Ochoa A, et al. Systematized review of the literature on postoperative nausea and vomiting. J Anesth Crit Care Open Access. 2023;15(3):101–7.

[22] Horn CC, Wallisch WJ, Homanics GE, Williams JP. Pathophysiological and neurochemical mechanisms of postoperative nausea and vomiting. Eur J Pharmacol. 2014;722:55–66. 24495419 10.1016/j.ejphar.2013.10.037 PMC3915298

[23] Simon N, Franchitto N, Rolland B. Pharmacokinetic Studies of Baclofen Are Not Sufficient to Establish an Optimized Dosage for Management of Alcohol Disorder. Front Psychiatry. 2018;9:485. 30349489 10.3389/fpsyt.2018.00485 PMC6186984

[24] Agarwal SK, Kriel RL, Cloyd JC, Coles LD, Scherkenbach LA, Tobin MH, et al. A pilot study assessing pharmacokinetics and tolerability of oral and intravenous baclofen in healthy adult volunteers. J Child Neurol. 2015;30(1):37–41. 25028414 10.1177/0883073814535504

[25] Schmitz NS, Krach LE, Coles LD, Mishra U, Agarwal SK, Cloyd JC et al. A randomized dose escalation study of intravenous baclofen in healthy volunteers: clinical tolerance and pharmacokinetics. PMR.2017;9(8):743–750. 10.1016/j.pmrj.2016.11.002 27867020

[26] Shellenberger MK, Groves L, Shah J, Novack GD. A controlled pharmacokinetic evaluation of tizanidine and baclofen at steady state. Drug Metab Dispos. 1999;27(2):201–4. 9929503

[27] Grigoras IF, Geist E, Johnstone A, Clarke WT, Emir U, Nettekoven C, et al. Baclofen, a GABAB receptor agonist, impairs motor learning in healthy people and changes inhibitory dynamics in motor areas. Imaging Neurosci (Camb). 2025;3:1–15. 10.1162/IMAG.a.979 PMC12580813 41190329

[28] Suzuki T, Nurrochmad A, Ozaki M, Khotib J, Nakamura A, Imai S, et al. Effect of a selective GABAB receptor agonist baclofen on the µ-opioid receptor agonist-induced antinociceptive, emetic and rewarding effects. Neuropharmacology. 2005;49(8):1121–31. 16095635 10.1016/j.neuropharm.2005.06.009

[29] Kadota T, Kakuta N, Horikawa YT, Tsutsumi R, Oyama T, Tanaka K, et al. Plasma substance P concentrations in patients undergoing general anesthesia: an objective marker associated with postoperative nausea and vomiting. JA Clin Rep. 2016;2(1):4–9. 29497664 10.1186/s40981-016-0034-9 PMC5818727

[30] Yamashita K, Abe T, Hayata Y, Hirose T, Hiraga S, Fukuba R, et al. Copeptin concentration following cardiac surgery as a prognostic marker of postoperative acute kidney injury: a prospective cohort study. J Thorac Disease. 2020;12(11):6609–17. 33282362 10.21037/jtd-20-2323 PMC7711377

[31] Hou Y, Liang H, Fan C, Feng Y. 5-Hydroxytryptamine and postoperative nausea and vomiting after microvascular decompression surgery. J Clin Neurosci. 2023;116:27–31. 37597331 10.1016/j.jocn.2023.08.010

[32] Garcia-Recio S, Gascón P. Biological and pharmacological aspects of the NK1-receptor. Biomed Res Int. 2015;2015(1):1–14. 10.1155/2015/495704 PMC4573218 26421291

[33] Lisowska B, Siewruk K, Lisowski A. Substance P and acute pain in patients undergoing orthopedic surgery. PLoS ONE. 2016;11(1):1–11. 10.1371/journal.pone.0146400 PMC4701134 26731421

[34] Lim BG, Choi SS, Jeong YJ, Kim YC, Park KU, Lee DK, et al. The relationship between perioperative nausea and vomiting and serum serotonin concentrations in patients undergoing cesarean section under epidural anesthesia. Korean J Anesthesiol. 2014;67(6):384–90. 25558338 10.4097/kjae.2014.67.6.384 PMC4280475

[35] Gosling P. Salt of the earth or a drop in the ocean? A pathophysiological approach to fluid resuscitation. Emerg Med J. 2003;20(4):306–15. 12835337 10.1136/emj.20.4.306 PMC1726159

[36] Nguyen NT, Wolfe BM. The Physiologic Effects of Pneumoperitoneum in the Morbidly Obese. Ann Surg. 2005;241(2):219–26. 15650630 10.1097/01.sla.0000151791.93571.70 PMC1356906

[37] Fromm GH. Baclofen as an adjuvant analgesic. J Pain Symptom Manage. 1994;9(8):500–9. 7852758 10.1016/0885-3924(94)90111-2

[38] Yomiya K, Matsuo N, Tomiyasu S, Yoshimoto T, Tamaki T, Suzuki T, et al. Baclofen as an adjuvant analgesic for cancer pain. Am J Hosp Palliat Med. 2009;26(2):112–8. 10.1177/1049909108327968 19114602

[39] Farishta A, Iancau A, Janis JE, Joshi GP. Use of Muscle Relaxants for Acute Postoperative Pain: A Practical Review. Plast Reconstr Surg. 2024;12(7):59–86. 10.1097/GOX.0000000000005938 PMC11216677 38957722

[40] Arabpour E, Khoshdel S, Akhgarzad A, Abdi M, Tabatabaie N, Alijanzadeh D, et al. Baclofen as a therapeutic option for gastroesophageal reflux disease: A systematic review of clinical trials. Front Med. 2023;10:1–21. 10.3389/fmed.2023.997440 PMC9981648 36873860

[41] Ertzgaard P, Campo C, Calabrese A. Efficacy and safety of oral baclofen in the management of spasticity: A rationale for intrathecal baclofen. J Rehabil Med. 2017;49(3):193–203. 28233010 10.2340/16501977-2211

[42] Addolorato G, Leggio L. Safety and Efficacy of Baclofen in the Treatment of Alcohol-Dependent Patients. Curr Pharm Des. 2010;16(19):2113–7. 20482507 10.2174/138161210791516440

[43] Nemeth BA, Montero RJ, Halanski MA, Noonan KJ. Epidural Baclofen for the Management of Postoperative Pain in Children with Cerebral Palsy. J Pediatr Orthop. 2015;35(6):571–5. 26251959 10.1097/BPO.0000000000000329

[44] Huh YJ, Kim DJ. Enhanced Recovery after Surgery in Bariatric Surgery. J Metab Bariatr Surg. 2021;10(2):47–54. 36683671 10.17476/jmbs.2021.10.2.47 PMC9847637

[45] Bhat S, Periasamy S, Arun M. Efficacy of Baclofen on Sequelae of Impacted Mandibular Third Molar Surgery. J Res Med Dent Sc. 2020;8(7):246–52.

[46] Zhang Q, Lehmann A, Dent J, Holloway RH. Control of transient lower esophageal sphincter relaxations and reflux by baclofen in GERD. Aliment Pharmacol Ther. 2002;16:1049–56.

[47] Frost EA. Differential diagnosis of delayed awakening from general anesthesia: A review. Middle East J Anaesthesiol. 2014;22(6):537–48. 25668997

[48] Zelcer J, Wells DG. Anaesthetic-related recovery room complications. Anaesth Intensive Care. 1987;15(2):168–174. 10.1177/0310057X8701500209 3605566

